# Supplementary material for: Quantitative trait loci mapping of gene expression and chromatin accessibility in primary fibroblasts reveals shared allelic effects between Latin American and European ancestries
Source: BMC Genomics. 2026 Jun 24;27:742. doi: 10.1186/s12864-026-13065-3 (PMC13551787; doi:10.1186/s12864-026-13065-3)
Supplement: Supplementary file 1 [file 12864_2026_13065_MOESM1_ESM.zip › updated Fibroblast Manuscript Figures and Tables/FigureS6.pdf]

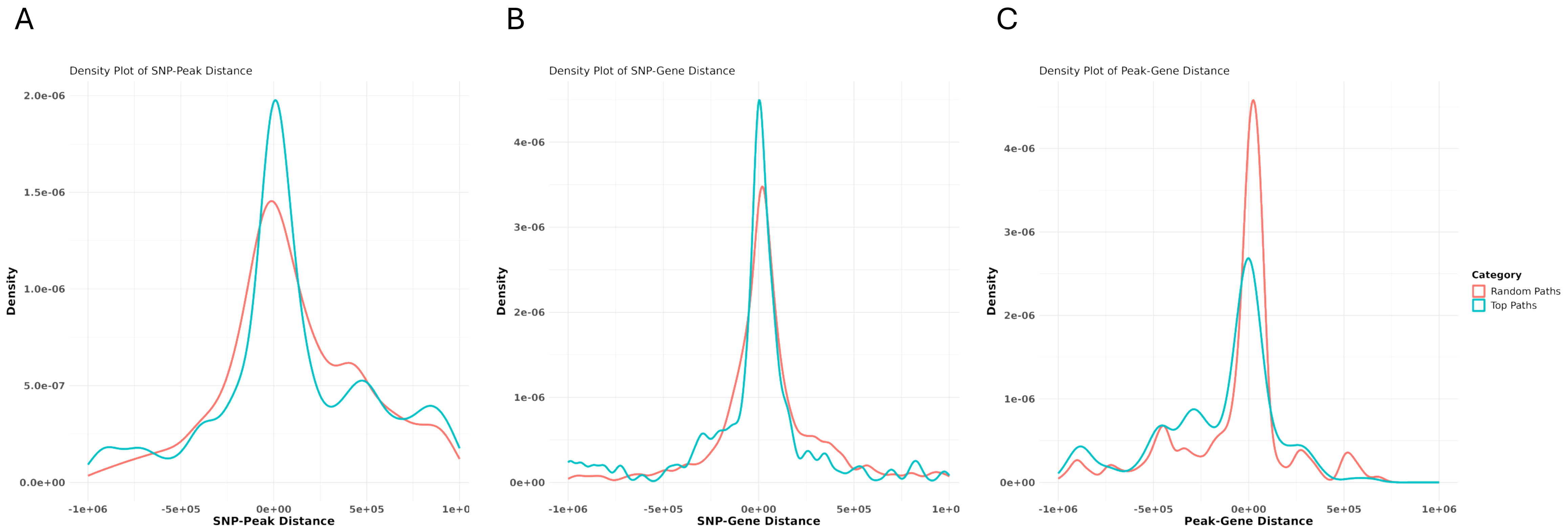
A
Density Plot of SNP-Peak Distance
2.0e-06
1.5e-06
1.0e-06
5.0e-07
0.0e+00
Density
-1e+06
-5e+05
0e+00
5e+05
1e+0
SNP-Peak Distance
B
Density Plot of SNP-Gene Distance
4e-06
3e-06
2e-06
1e-06
0e+00
Density
-1e+06
-5e+05
0e+00
5e+05
1e+0
SNP-Gene Distance
C
Density Plot of Peak-Gene Distance
4e-06
3e-06
2e-06
1e-06
0e+00
Density
-1e+06
-5e+05
0e+00
5e+05
1e+06
Peak-Gene Distance
Category
Random Paths
Top Paths
